# Hydrogen sulfide ameliorates subarachnoid hemorrhage-induced neuronal apoptosis *via* the ROS-MST1 pathway

**DOI:** 10.18632/oncotarget.20569

**Published:** 2017-08-28

**Authors:** Ligen Shi, Jianwei Lei, Hangzhe Xu, Jingwei Zheng, Yan Wang, Yucong Peng, Jun Yu, Jianmin Zhang

**Affiliations:** ^1^ Department of Neurosurgery, Second Affiliated Hospital, School of Medicine, Zhejiang University, Hangzhou, Zhejiang, China; ^2^ Brain Research Institute, Zhejiang University, Hangzhou, Zhejiang, China; ^3^ Collaborative Innovation Center for Brain Science, Zhejiang University, Hangzhou, Zhejiang, China

**Keywords:** hydrogen sulfide, MST1, neuronal apoptosis, subarachnoid hemorrhage, early brain injury, Pathology Section

## Abstract

**Background:**

Hydrogen sulfide (H_2_S) has shown a neuroprotective role in several cerebrovascular diseases. This study aimed to explore the underlying mechanisms of H_2_S in early brain injury after subarachnoid hemorrhage (SAH).

**Methods:**

One hundred seventy-seven male Sprague-Dawley rats were employed in this study. Sodium hydrosulfide (NaHS), a donor of H_2_S, was injected intraperitoneally at 60 min after SAH was induced by endovascular perforation. Western blot analysis determined the expression of several proteins of interest, and an immunofluorescence assay was used to examine neuronal apoptosis.

**Results:**

Exogenous NaHS markedly improved neurological scores, attenuated brain edema, and ameliorated neuronal apoptosis at 24 h after SAH induction. The underlying mechanisms of H_2_S in ameliorating neuronal apoptosis might be executed through inhibition of the activity of mammalian sterile 20-like kinase 1 (MST1) protein. Western blot analysis demonstrated that exogenous NaHS decreased cleaved MST1 (cl-MST1) while increasing full-length MST1 expression. This anti-apoptotic effect of H_2_S could be reversed by chelerythrine, which could activate MST1 via caspase-dependent cleavage.

**Conclusions:**

Exogenous NaHS, as a donor of H_2_S, could ameliorate early brain injury after SAH by inhibiting neuronal apoptosis by reducing the activity of the MST1 protein.

## INTRODUCTION

Aneurysmal subarachnoid hemorrhage (SAH) is a common and severe subtype of hemorrhagic stroke with a high mortality [1]. Early brain injury (EBI), which occurs within the first 72 hours following the aneurysm rupture, is considered to be the main aspect that contributes to an unfavorable outcome [2]. Among multiple complex mechanisms of EBI after SAH, apoptosis is regarded as one of the most crucial factors that may be associated with delayed neurological deterioration and poor long-term outcomes [3]. Although numerous studies explored the mechanism of apoptosis, none of the possible anti-apoptotic agents has been demonstrated to be ideal in subsequent clinical trials [4].

Oxidative stress, primarily generated by oxyhemoglobin stimulation, has been considered to be an intense pro-apoptotic factor in the pathogenesis of SAH-induced EBI [5]. Excessive reactive oxygen species (ROS) could initiate the activation of mammalian sterile 20-like kinase 1 (MST1) [6], which is a crucial serine-threonine kinase that belongs to a critical component of the Hippo signaling pathway [7]. MST1 has been shown to have important functions in apoptotic cell death [8]. Previous studies have indicated that oxidative stress activates MST1 and simultaneously cleaves MST1 to produce a 36 kDa N-terminal constitutively active fragment (cl-MST1) [9]. This cl-MST1 has a 10-fold higher activity than the full-length Mst1 kinase [9]. Subsequently, the cl-MST1 is transferred into the nucleus and phosphorylates several histones, inducing neuronal cell apoptosis [10]. Agents that can inhibit the ROS-MST1-induced cell apoptosis pathway may be an alternative anti-apoptotic agent for EBI after SAH.

Hydrogen sulfide (H_2_S) may be a potential excellent anti-apoptotic agent for SAH-induced EBI. H_2_S is expressed at relatively high levels in astrocytes, neurons, and microglia [11] and can rapidly diffuse through the cell membrane to exert its effects within seconds due to its high lipophilicity [12]. Previous studies have indicated that H_2_S could inhibit oxidative stress-induced primary rat cortical neuronal apoptosis [13]. Moreover, H_2_S protects hippocampal neuronal cells from hypoxia-induced apoptosis by blocking an ROS-activated Ca^2+^ signaling pathway [14]. Recently, the neuroprotective effects of H_2_S in SAH have been increasingly recognized [15]. H_2_S production was rapidly down-regulated in the rat brain after the occurrence of SAH [15]. Meanwhile, the protein levels of two major catalytic enzymes of H_2_S that are highly expressed in the brain, cystathionine-β-synthase (CBS) and 3-mercaptopyruvate sulfur transferase (3MST), were also observed to be down-regulated in SAH rats [15]. Treatment with sodium hydrosulfide (NaHS), a donor of H_2_S, restored the brain content of H_2_S and the protein levels of CBS and 3MST [15]. Moreover, H_2_S has been shown to protect the blood brain barrier, inhibit apoptosis, reduce brain edema, and improve neurological outcomes after SAH [16, 17]. However, the underlying anti-apoptotic mechanism of H_2_S remains largely unknown.

Therefore, the present study tested the hypothesis that oxidative stress after SAH may induce the cleavage of MST1 to produce cl-MST1, resulting in neuronal cell apoptosis, and that exogenous NaHS can increase the brain content of H_2_S to inhibit MST1-induced neuronal cell apoptosis.

## RESULTS

### Overview of body weight, blood pressure, temperature, and mortality in both SAH and sham rats

No significant differences were observed in body weight, mean arterial blood pressure, or temperature among any of the experimental groups (data not shown). No rats in the sham group died (0 of 30 rats). Total mortality of the SAH rats was 21.09% (31 of 147 rats) within 24 hours after SAH induction. No significant difference was observed for mortality among the groups that had undergone the surgical operation: mortality was 21.95% (18 of 82 rats) in the SAH group, 19.57% (9 of 46 rats) in the SAH + H_2_S group, and 21.05% (4 of 19 rats) in the SAH + H_2_S + chelerythrine group. Eight SAH rats were excluded from this study due to mild bleeding (SAH grade score < 8).

### MST1 protein was mainly expressed in the cytoplasm of neuronal cells

**Figure 1:**
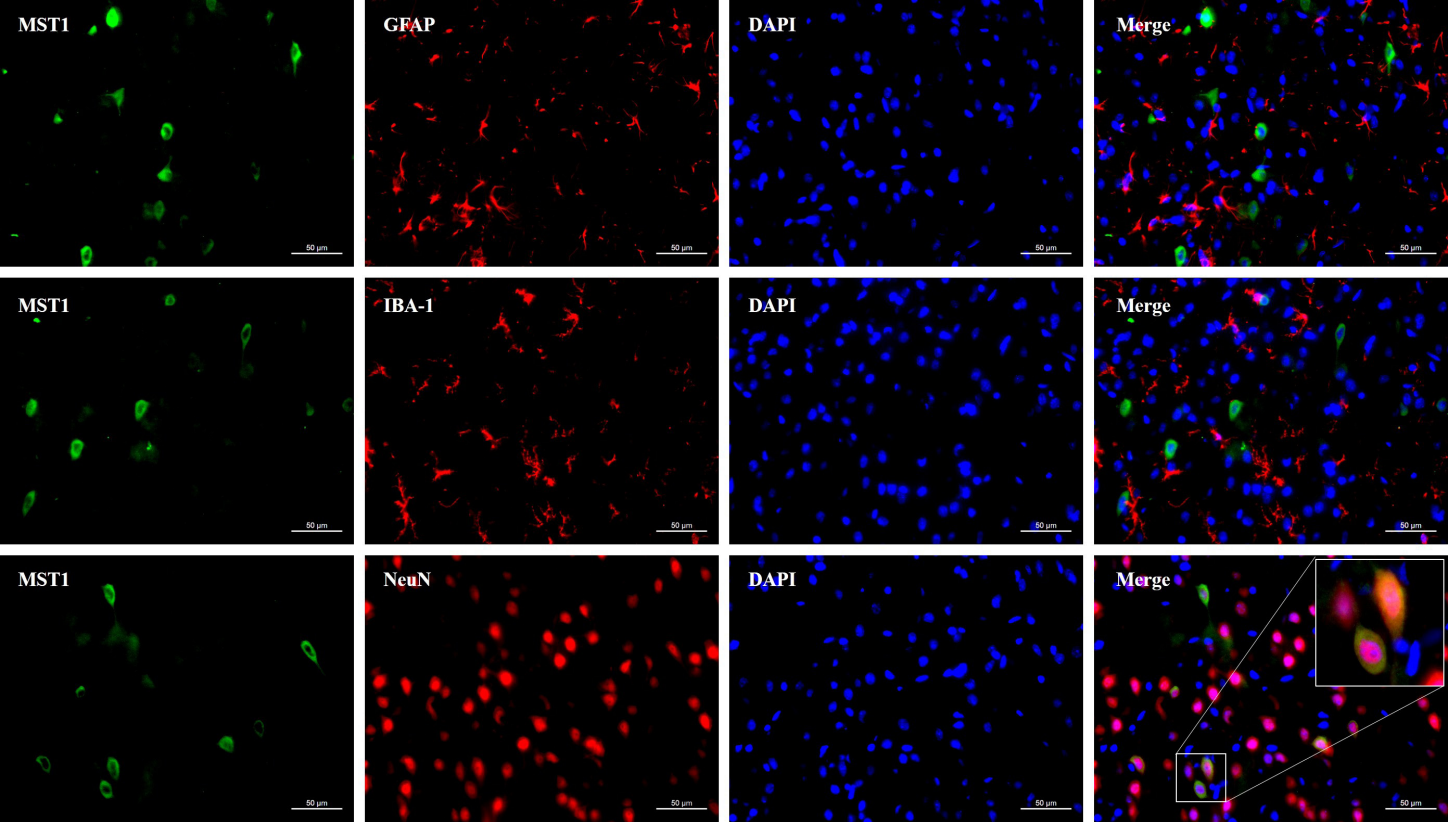
The MST1 protein was primarily expressed in the cytoplasm of neurons Representative images of immunofluorescence staining showed the expression of MST1 with NeuN, GFAP, and IBA-1 at 24 h after subarachnoid hemorrhage (*n* = 6). Scale bar: 20 μm.

Double immunofluorescence staining was performed to detect the location of MST1 in the ipsilateral cortex around the perforation area, which demonstrated that MST1 was co-expressed with NeuN in the cytoplasm (Figure 1). In addition, little MST1 was observed to co-express with GFAP or IBA-1 (Figure 1). These data indicated that MST1 was mainly expressed in the cytoplasm of the neuronal cells.

### MST1 expression was downregulated after SAH

**Figure 2:**
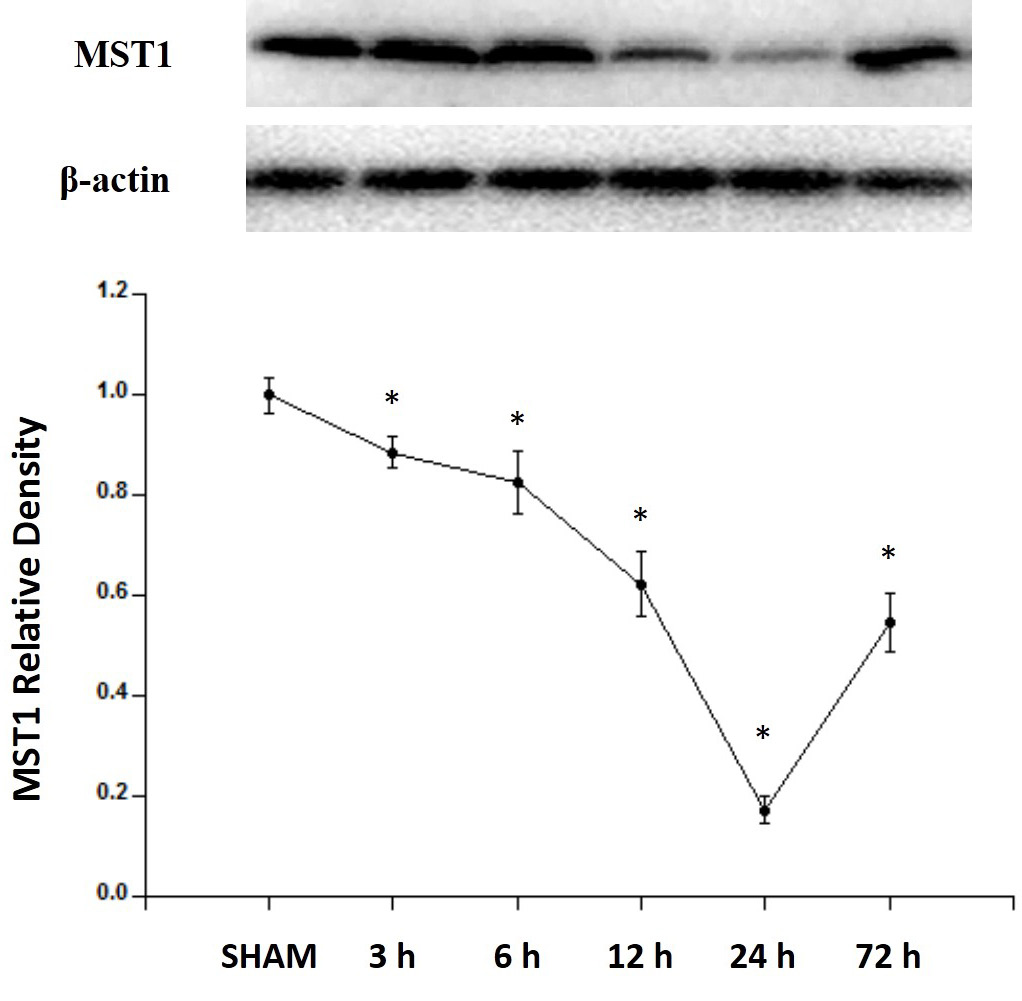
Expression profiles of MST1 in SAH rats Western blot assay showing the endogenous expression profiles of MST1 in sham and SAH rats for 3, 6, 12, 24, and 72 h (*n* = 6 for each time point). Error bars represent the mean ± standard error (SEM). * *P* < 0.05 versus sham group.

The expression of MST1 showed a significant decrease at 3 h, gradually decreased to reach its lowest level at 24 h, and remained at a low level at 72 h after SAH induction (Figure 2).

### NaHS treatment alleviated brain edema and improved neurological function

**Figure 3:**
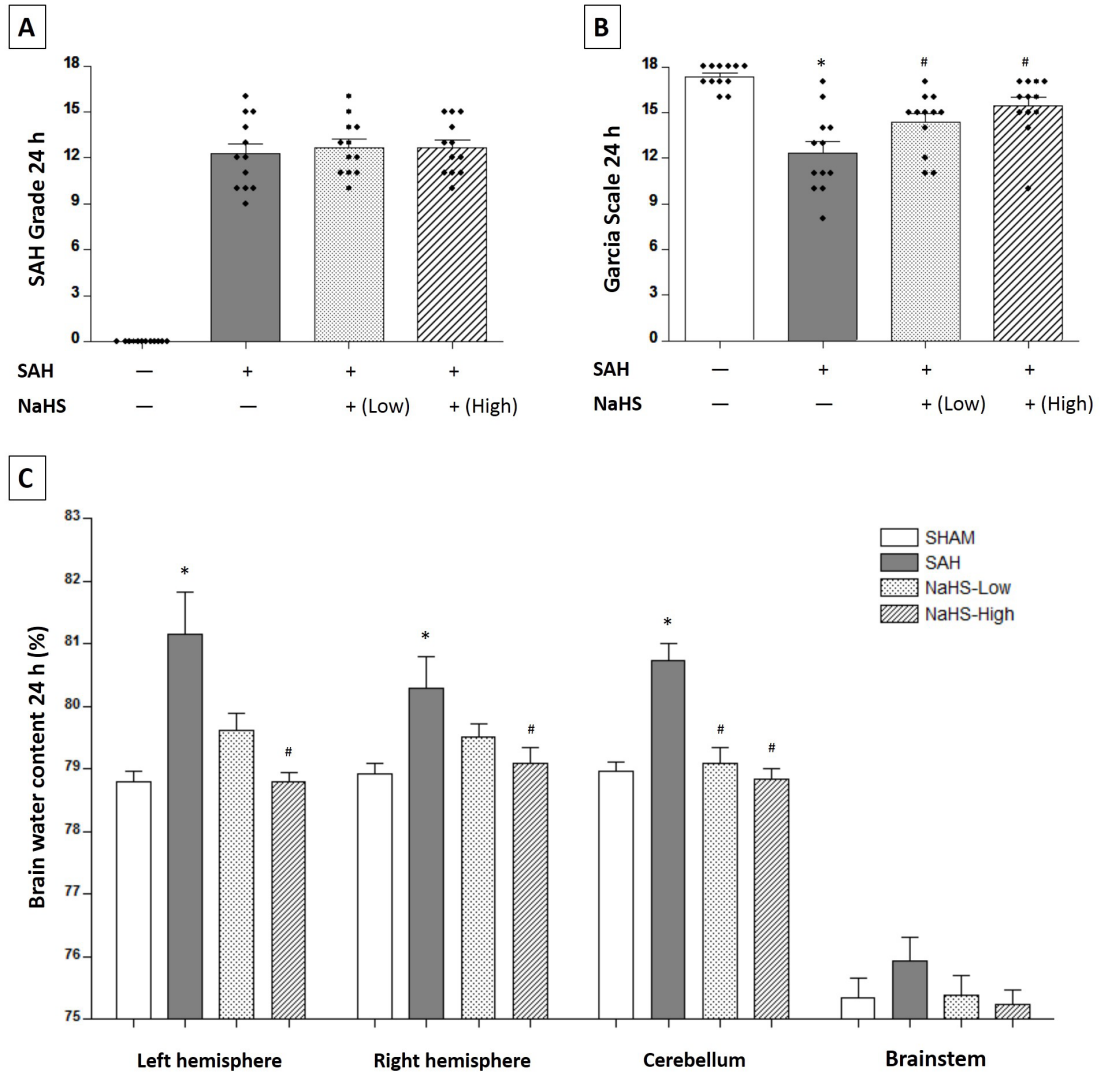
Both low and high dosages of exogenous NaHS alleviated brain edema and improved neurological function at 24 hours after SAH induction **A.** SAH grade (*n* = 12 for each group): a similar SAH grade was observed in the SAH group and the NaHS groups. **B.** Garcia test (*n* = 12 for each group): Garcia score decreased at 24 hours after SAH induction. Both low and high dosages of exogenous NaHS treatment significantly increased the Garcia score compared with the SAH group. **C.** Brain water content (*n* = 6 for each group): a low dosage of NaHS treatment significantly decreased brain water content in the cerebellum. A high dosage of NaHS therapy significantly decreased brain water content in the left hemisphere, the right hemisphere, and the cerebellum. Error bars represent the mean ± standard error (SEM). *P < 0.05 versus sham group; #*P* < 0.05 versus SAH group.

Two dosages of NaHS (1.4 mg/kg and 5.6 mg/kg) were injected intraperitoneally at 1 hour after SAH induction. There were no significant differences in the SAH scores among the SAH rats receiving vehicle (12.25 ± 0.6756), a low dosage of NaHS (12.67 ± 0.5270), or a high dosage of NaHS (12.67 ± 0.5125) at 24 hours (*P* > 0.05, Figure 3A). SAH rats receiving vehicle showed a significantly worse Garcia score at 24 hours compared with the sham group (12.33 ± 0.7621 vs. 17.33 ± 0.2247, *P* < 0.01; Figure 3B). Both low and high dosages of NaHS showed significant improvements in neurological function (Garcia score: 14.33 ± 0.5685 for the low dosage of NaHS, 15.42 ± 0.5702 for the high dosage of NaHS, vs. 12.33 ± 0.7621 for the SAH rats, *P* < 0.05; Figure 3B). In addition, the SAH rats had a higher ratio of brain water content in the left hemisphere (81.16 ± 0.0751 vs. 78.79 ± 0.1757, *P* < 0.01; Figure 3C), the right hemisphere (80.29 ± 0.4951 vs. 78.91 ± 0.1731, *P* < 0.05; Figure 3C), and the cerebellum (80.74 ± 0.2688 vs.78.97 ± 0.14447, *P* < 0.05; Figure 3C) than rats in the sham group. The low dosage of NaHS alleviated brain edema in the cerebellum (79.08 ± 0.2658 vs. 80.74 ± 0.2688, *P* < 0.05; Figure 3C), and the high dosage of NaHS alleviated brain edema in the left hemisphere (78.80 ± 0.1381 vs. 81.16 ± 0.0751, *P* < 0.01; Figure 3C), the right hemisphere (79.09 ± 0.2551 vs. 80.29 ± 0.4951, *P* < 0.05; Figure 3C), and the cerebellum (78.83 ± 0.1795 vs. 80.74 ± 0.2688, *P* < 0.01; Figure 3C).

### Both low and high dosages of NaHS decreased neuronal apoptosis

**Figure 4:**
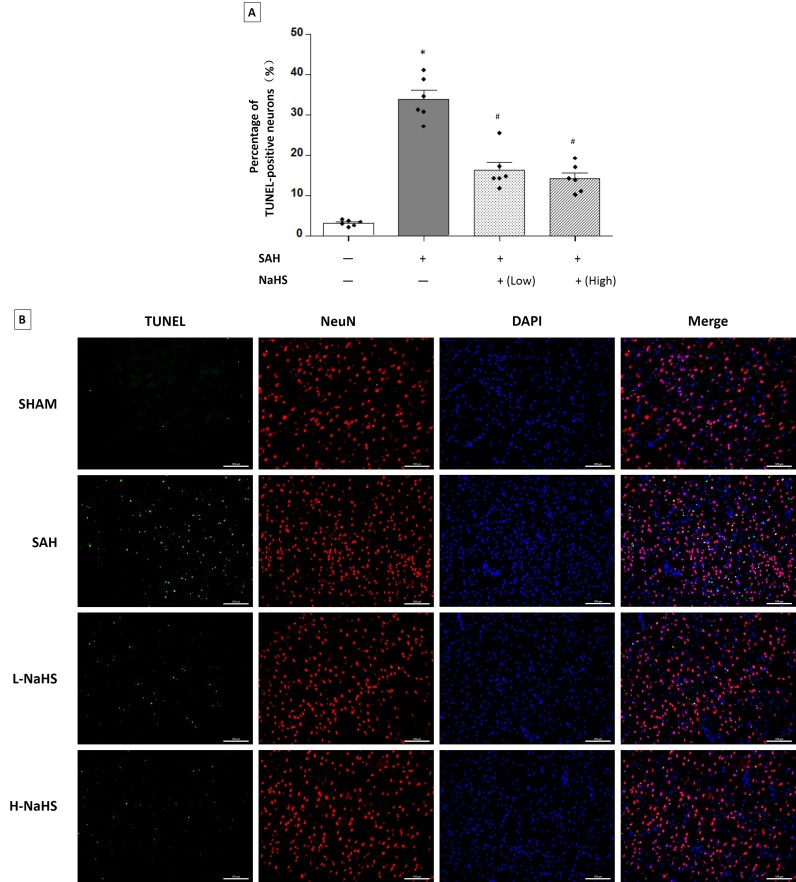
Both low and high dosages of exogenous NaHS decreased neuronal cell apoptosis **A.** Neuronal cell apoptosis (*n* = 6 for each group): a significant increase in neuronal cell apoptosis was observed in the SAH group. Both low and high dosages of NaHS treatment significantly decreased neuronal cell apoptosis compared with the SAH group. Error bars represent the mean ± standard error (SEM). *P < 0.05 versus sham group; #*P* < 0.05 versus SAH group. **B.** Representative images of immunofluorescence staining show the TUNEL-positive neuronal cells (*n* = 6 for each group). Scale bar: 100 μm.

TUNEL staining detected neuronal apoptosis at 24 hours after SAH induction. SAH rats showed a higher percentage of TUNEL-positive neurons compared with sham rats (33.96 ± 2.144% vs. 3.077 ± 0.2996%, *P* < 0.01; Figure 4). The SAH rats receiving either low or high dosages of NaHS showed a significant inhibition of neuronal apoptosis (TUNEL-positive neurons %: 16.29 ± 1.964% for the low dosage of NaHS, 14.28 ± 1.407% for the high dosage of NaHS, and 33.96 ± 2.144% for the SAH rats, *P* < 0.05; Figure 4).

### Chelerythrine abolished the effects of NaHS on inhibiting SAH-induced apoptosis

**Figure 5:**
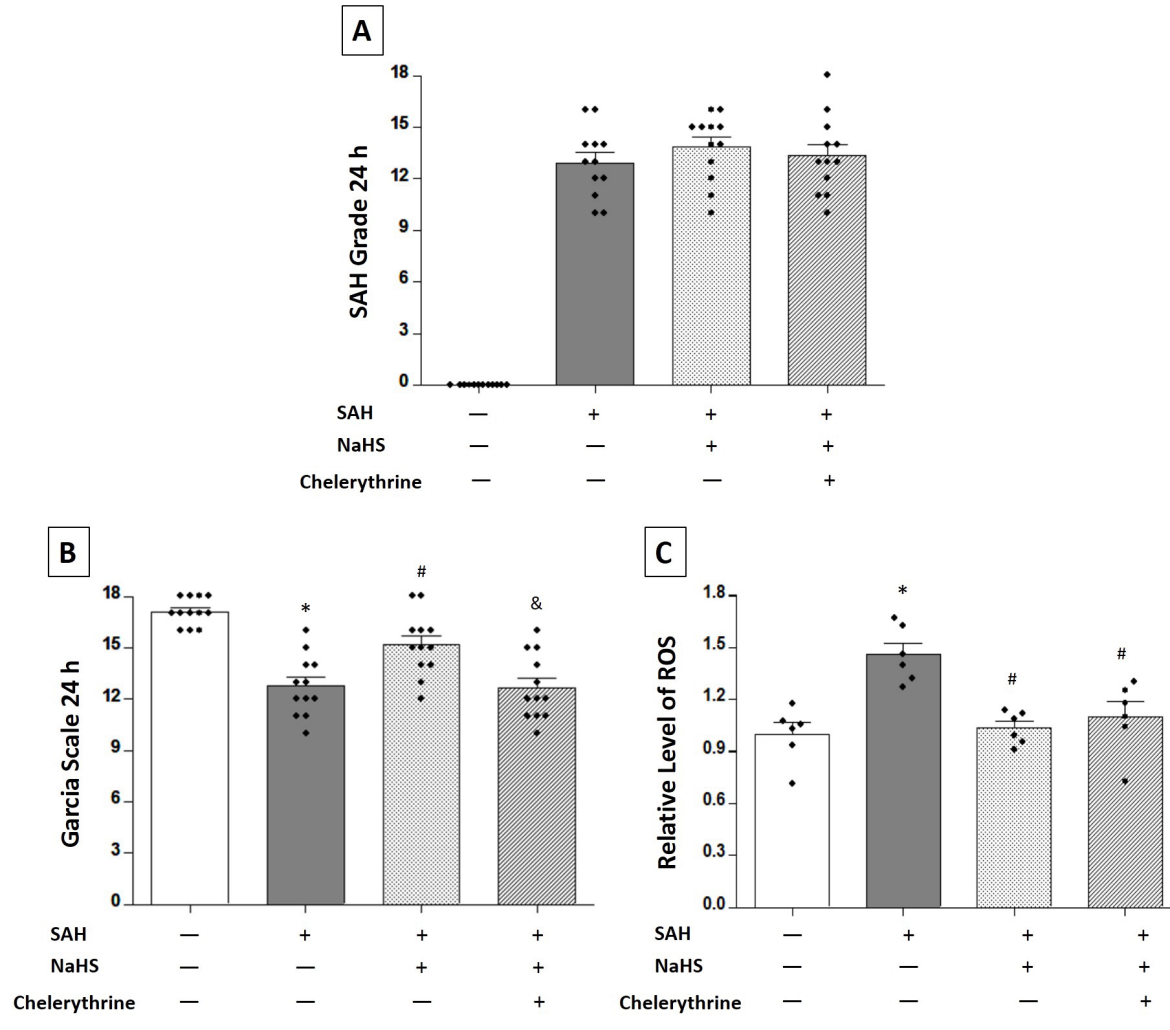
Exogenous NaHS reduced SAH-induced ROS levels in the brain tissues For these experiments, the rats were randomly divided into four groups: sham group, SAH rats receiving vehicle, SAH rats receiving NaHS, and SAH rats receiving both NaHS and chelerythrine. **A.** SAH grade (*n* = 12 for each group): a similar SAH grade was observed in the SAH group and the NaHS groups. **B.** Garcia test (*n* = 12 for each group): Garcia score decreased at 24 hours after SAH induction. Exogenous NaHS treatment significantly increased the Garcia score compared with the SAH group, while chelerythrine remarkably reversed the improvement of neurological function by NaHS treatment. **C.** ROS level (*n* = 6 for each group): exogenous NaHS treatment significantly decreased the SAH-induced high level of ROS in the brain tissues, while chelerythrine had no effect on the ROS level. Error bars represent the mean ± standard error (SEM). *P < 0.05 versus sham group; &*P* < 0.05 versus SAH+NaHS group.

Chelerythrine, as an MST1 agonist that acts via caspase-dependent cleavage, was applied immediately after SAH induction. No significant differences were observed in the SAH scores among the SAH rats receiving vehicle (12.92 ± 0.5833), NaHS (13.83 ± 0.5618), or NaHS plus chelerythrine (13.33 ± 0.6552) at 24 hours (*P* > 0.05, Figure 5A). SAH rats receiving vehicle had worse neurological function (12.75 ± 0.5094 vs. 17.08 ± 0.2289, *P* < 0.01; Figure 5B), but NaHS improved this SAH-induced neurologic deficit (15.17 ± 0.5198 vs. 12.75 ± 0.5094, *P* < 0.01; Figure 5B), while chelerythrine reversed the neuroprotective effect of NaHS (12.67 ± 0.5551 vs. 15.17 ± 0.5198, *P* < 0.01; Figure 5B). The SAH group showed a significant increase in the level of ROS compared with the sham group (1.460 ± 0.06603 vs. 1.000 ± 0.06550, *P* < 0.01; Figure 5C). NaHS treatment could markedly reduce the level of ROS (1.037 ± 0.03863 vs. 1.460 ± 0.06603, *P* < 0.01; Figure 5C), and chelerythrine had no effect on the level of ROS (1.102 ± 0.08424 vs. 1.037 ± 0.03863, *P* > 0.05; Figure 5C).

**Figure 6:**
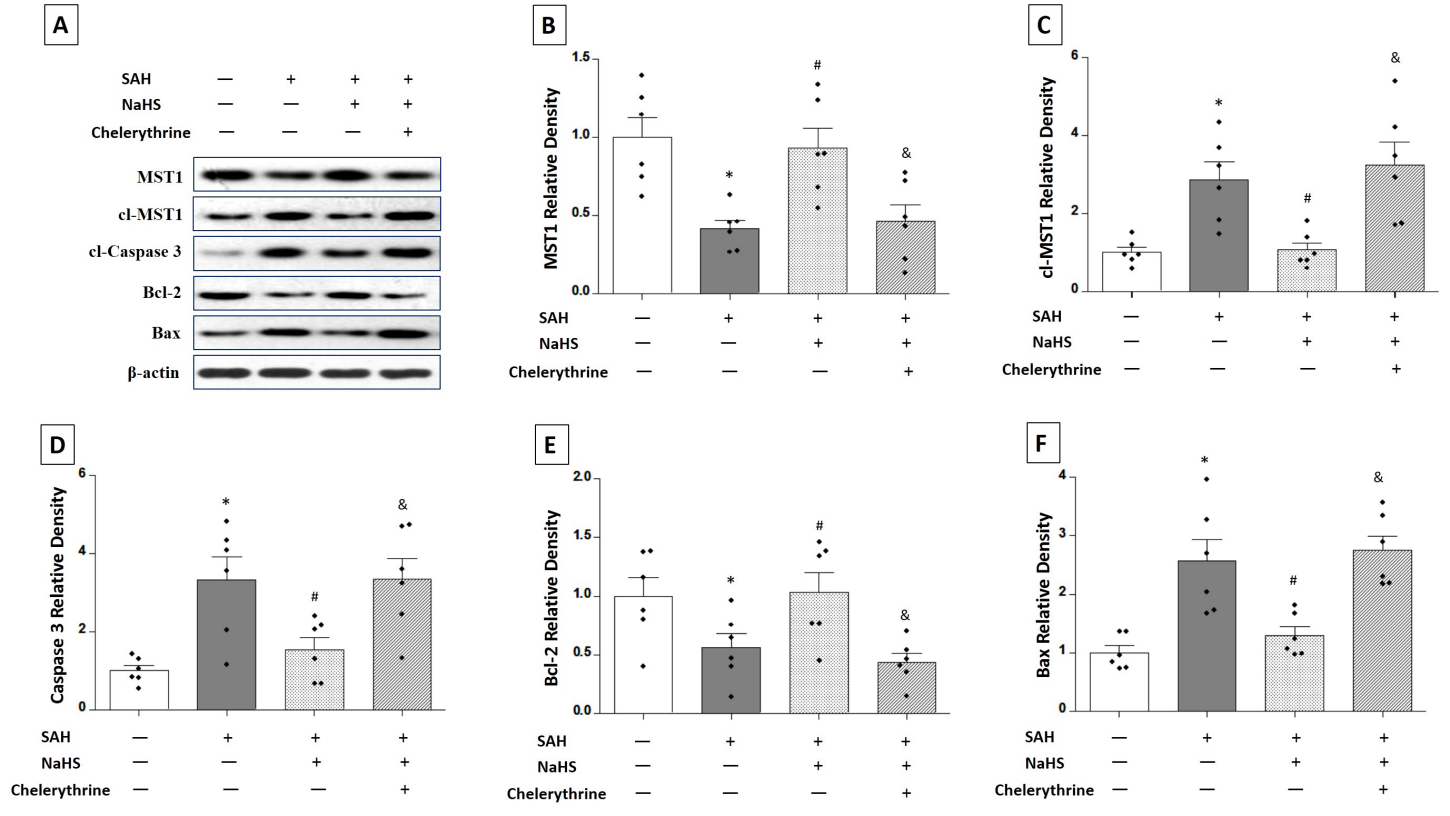
Western blots showing the expression of MST1, cl-MST1, caspase 3, Bcl-2, and Bax in response to NaHS treatment and chelerythrine administration **A.** Rats were randomly divided into four groups: sham group, SAH rats receiving vehicle, SAH rats receiving NaHS, and SAH rats receiving both NaHS and chelerythrine. The expression of MST1, cl-MST1, caspase 3, Bcl-2, and Bax was detected by western blots. The results showed that the expression of MST1 **B.** and Bcl-2 **E.** was increased by NaHS therapy compared with the SAH group, and reversed after using chelerythrine. In contrast, the expression of cl-MST1 **C.**, caspase 3 **D.**, and Bax **F.** was decreased by NaHS therapy compared with the SAH group, and reversed after using chelerythrine. Error bars represent the mean ± standard error (SEM). *P < 0.05 versus sham group; #*P* < 0.05 versus the SAH group. &*P* < 0.05 versus SAH+NaHS group.

Western blot analysis was performed to detect the expression of the target proteins, including MST1, cl-MST1, caspase 3, Bcl-2, and Bax (Figure 6A). The SAH rats receiving vehicle showed a 58.62% decrease in the expression of MST1 (*P* < 0.01; Figure 6B), a 287.1% increase in the expression of cl-MST1 (*P* < 0.01; Figure 6C), a 334.0% increase in the expression of caspase 3 (*P* < 0.01; Figure 6D), a 43.64% decrease in the expression of Bcl-2 (*P* < 0.05; Figure 6E), and a 256.4% increase in the expression of Bax (*P* < 0.05; Figure 6F). NaHS treatment up-regulated the expression of MST1 (0.9314 ± 0.1256 vs. 0.4138 ± 0.05577, *P* < 0.01; Figure 6B) and Bcl-2 (1.029 ± 0.1723 vs. 0.5636 ± 0.1185, *P* < 0.05; Figure 6E) and down-regulated the expression of cl-MST1 (1.061 ± 0.1868 vs. 2.871 ± 0.4504, *P* < 0.01; Figure 6C), caspase 3 (1.544 ± 0.3138 vs. 3.340 ± 0.5844, *P* < 0.05; Figure 6D), and Bax (1.292 ± 0.1489 vs. 2.564 ± 0.3750, *P* < 0.05; Figure 6F). Chelerythrine could reverse the effect of NaHS on the expression of MST1 (0.4633 ± 0.1058 vs. 0.9314 ± 0.1256, *P* < 0.05; Figure 6B), cl-MST1 (3.250 ± 0.5864 vs. 1.061 ± 0.1868, *P* < 0.01; Figure 6C), caspase 3 (3.342 ± 0.5415 vs. 1.544 ± 0.3138, *P* < 0.05; Figure 6D), Bcl-2 (0.4370 ± 0.07672 vs. 1.029 ± 0.1723, *P* < 0.05; Figure 6E), and Bax (2.749 ± 0.2505 vs. 1.292 ± 0.1489, *P* < 0.01; Figure 6F).

## DISCUSSION

The present study was designed to explore the effect of H_2_S on inhibiting ROS-MST1-induced neuronal apoptosis in SAH rats. The results showed that MST1 was primarily expressed in the cytoplasm of neuronal cells. After suffering SAH, MST1 expression decreased immediately and reached its lowest level at 24 hours. Meanwhile, the high levels of ROS induced by SAH stimulated the cleavage of MST to cl-MST1, which up-regulated the expression of Bax and down-regulated the expression of Bcl-2, leading to neuronal apoptosis. Exogenous NaHS could inhibit the high level of ROS induced by SAH, then down-regulated the expression of cl-MST1 to inhibit neuronal apoptosis, and finally improved brain edema and the neurological deficits.

MST1 is a 487-amino acid protein with a molecular mass of 59-63 kDa [18] that is expressed ubiquitously in mammalian cells [19]. Double immunofluorescence staining results showed that MST1 was primarily expressed in neurons but was barely observed in astrocytes and microglia of SAH rats. This finding was inconsistent with previous studies in which MST1 was reported to exist in astrocytes [20] and microglia [21]. This may be due to different brain tissues that were used in different studies. The present study focused on brain tissues around the basal ganglia area under SAH induction, which might cause a decreased expression in the MST1 protein in astrocytes and microglia. In addition, previous studies showed that MST1 was exclusively located in the cytoplasm under resting conditions but showed partial transfer to the nucleus in the presence of a destructive stimulus [9]. In the present study, we observed that MST1 was primarily expressed in the cytoplasm, which was consistent with previous findings.

Previous literature has indicated that oxyhemoglobin from erythrocytes in the subarachnoid blood clot could cause oxidative stress [22]. Consistent with these findings, the level of ROS significantly increased after SAH induction in the present study. In addition, MST1 could be activated by various pro-apoptotic stimuli and cellular stresses in a number of cell lines [23, 24]. In particular, exposure to oxidative stress strongly initiates the cleavage activation of MST1 to stimulate caspase-dependent apoptosis [6, 25]. Hence, we observed that the SAH-induced overexpression of ROS reduced the expression of MST1 and increased the expression of cl-MST1. Subsequently, the activated MST1 triggered the caspase-dependent apoptosis in which we observed the down-regulation of Bcl-2 and the up-regulation of Bax and caspase 3. However, the present study did not explore the exact mechanism of ROS activating MST1 to induce neuronal apoptosis. According to previous studies, neuronal cells that have suffered oxidative stress would first induce c-abl-dependent tyrosine phosphorylation. Subsequently, the activated c-abl promotes the transfer of MST1 into the nucleus, inducing neuronal cell apoptosis [26].

Endogenous H_2_S produced by CBS and 3MST in physiological conditions, which is localized to the mitochondria, can directly decrease the production of ROS and inhibit neuronal apoptosis [27]. NaHS, as a donor of H_2_S, has been proven to increase the content of H_2_S in brain and to enhance the activity of CBS and 3MST [15]. In the present study, exogenous NaHS reduced the level of ROS in the brain tissues of SAH rats. Meanwhile, NaHS inhibited the expression of cl-MST1 and caspase 3. To confirm whether NaHS reduced neuronal apoptosis by inhibiting the cleavage of MST1, we set up a parallel group of SAH rats receiving both NaHS and chelerythrine. The results indicated that chelerythrine, as an MST1 agonist that enhances caspase-dependent cleavage, reversed the anti-apoptotic effect of NaHS in SAH rats. Hence, H_2_S, as an antioxidant, scavenges the overloaded ROS to decrease the levels of cleaved-MST1, leading to the inhibition of neuronal apoptosis. In addition, NaHS treatment increased the levels of the anti-apoptotic protein Bcl-2 and reduced the levels of the pro-apoptotic protein Bax. These changes were also observed in previous studies that demonstrated that H_2_S could reduce the ratio of Bax/Bcl-2 in SAH rats [17]. Although previous studies have indicated that MST1 has no effect on the expression of Bcl-2 and Bax [28], MST1 could regulate the interaction between Bcl-2 and Bax [29]. Hence, we believe that these changes in Bcl-2 and Bax were also partially a consequence of MST1 modifications. In the present study, exogenous NaHS was administered to SAH rats at 1 hour after SAH induction, which made clinical use possible. Although previous studies demonstrated that high levels of H_2_S will act as a toxic molecule inhibiting mitochondrial respiration [30], both low and high levels of NaHS showed a significant improvement in brain edema and neurological function in the present study. We believe that NaHS (5.6 mg/kg via intraperitoneal administration) has significant efficacy and satisfactory safety. In addition, a placebo-controlled dose-escalation phase I clinical trial examined the safety of an α-sulfur oral formulation H_2_S prodrug in healthy and congestive heart failure patients [31], and demonstrated that exogenous H_2_S was well tolerated and safe in human patients [31]. Hence, we believe that exogenous H_2_S is a potential neuroprotective agent for clinical treatment to SAH patients. However, the present study only detected the effect of NaHS administered at 1 hour after SAH induction. Whether exogenous NaHS still works in a wider time window needs to be examined in further studies. In addition, it should be noted that the present study did not detect other apoptotic proteins or components of the Hippo pathway in the present study. There may be other pathways involved in the anti-apoptotic effect of H_2_S in reducing neuronal apoptosis.

Several limitations should be noted in the present study. First, the present study only explored the effect of NaHS on inhibiting MST1-induced neuronal apoptosis in EBI after SAH. We did not observe the long-term effect of NaHS on neurological function. We have completed the preparation of a study protocol for our next study to cover the limitations of this present study. Second, we did not detect the H_2_S contents in cerebrospinal fluid of SAH rats, although previous studies have proven that exogenous NaHS applied via intraperitoneal administration could increase the H_2_S contents in cerebrospinal fluid [15-17]. Third, chelerythrine is a potent protein kinase C inhibitor with several targets [32]. Hence, it is not a specific agonist of MST1. Although previous studies have demonstrated that chelerythrine could activate MST1 via caspase-dependent cleavage, the present study did not rule out the possibility that chelerythrine induced apoptosis through other pathways.

In conclusion, our study observed for the first time that the ROS-MST1-caspase pathway plays an important role in inducing neuronal apoptosis in EBI after SAH induction. Exogenous NaHS successfully inhibited this ROS-MST1-induced neuronal apoptosis, alleviated brain edema, and improved neurological function at 24 hours after SAH induction. Further studies should explore the long-term effect of H_2_S on neurological function recovery.

## MATERIALS AND METHODS

### Animals

All experiments followed *the Guide for the Care and Use of Laboratory Animals of the National Institutes of Health and Animal Research: Reporting of In Vivo Experiments (ARRIVE) guidelines*. The animal protocol was approved by the Institutional Ethics Committee of the Second Affiliated Hospital, Zhejiang University School of Medicine. One hundred seventy-seven male Sprague-Dawley rats (weight 300-320 g) were purchased from the Slac Laboratory Animal Company Limited *(Shanghai, China)*. All rats were housed in a facility with temperature- and humidity-controlled conditions with 12-h light/dark cycles. Animal body temperature was maintained at 37 °C.

### SAH model

An endovascular perforation SAH model was used as previously reported [33, 34]. Briefly, we used 40 mg/kg pentobarbital sodium to anesthetize the experimental rats via intraperitoneal injection. Subsequently, the left carotid artery and its branches were explored by microsurgical isolation. We inserted a sharpened 4-0 monofilament nylon suture into the internal carotid artery from the external carotid artery and pushed it forward until we felt the resistance from the bifurcation of the anterior and middle cerebral arteries. The nylon suture was then rapidly advanced approximately 1 mm to create SAH and then left motionless for 10 s. Similar surgical procedures were performed in sham rats without perforation. During the whole operative procedure, all of the experimental rats were maintained at 37 °C using a heating pad.

### Drug administration

An exogenous H_2_S donor, NaHS (Sigma-Aldrich, USA), was dissolved in sterile saline (25 μmol/L and 100 μmol/L) and administered to SAH rats via intraperitoneal injection at 1 hour after SAH induction. Sterile saline was administered as a vehicle control. Chelerythrine, as an MST1 agonist, was dissolved in dimethyl sulfoxide (1 mmol/L) and administered immediately via intracerebroventricular administration after SAH. Dimethyl sulfoxide was administered as a vehicle control.

### Experimental design

Before performing these experiments, we calculated sample size, determining that six rats per group should be suitable. We randomly assigned these rats into each group according to a randomized digital table. During the experiments, all authors, except for the principal investigator (JM Zhang), were blinded to the details of the study.

Three separate experiments were conducted in this study.

*Experiment I aimed to determine the expression of MST1 after SAH.* Western blots detected the expression of MST1 in sham and SAH rats at 3, 6, 12, 24, and 72 hours (*n* = 6 for each group). Double immunofluorescence staining examined the expression location of MST1 in astrocytes, neurons, and microglia of SAH rats (*n* = 6 for each group).

*Experiment II was performed to assess the effect of NaHS on early brain injury after SAH and to determine the optimal dosage for the subsequent mechanism exploration.* The rats were randomly divided into four groups: sham group, SAH+vehicle (0.9% sterile saline) group, SAH+NaHS low-dosage (1.4 mg/kg) group, and SAH+NaHS high-dosage (5.6 mg/kg) group. Neurobehavioral functions, brain water content, and neuronal apoptosis were evaluated at 24 hours after SAH induction. Twelve rats per group were used to assess neurobehavioral functions and were then separately sacrificed to evaluate brain water content (*n* = 6 for each group) and neuronal apoptosis (*n* = 6 for each group).

*Experiment III aimed to detect the mechanism of NaHS in inhibiting neuronal apoptosis after SAH.* Chelerythrine (1 mmol/L, 10 µl), as an MST1 agonist that acts via caspase-dependent cleavage, was given immediately via intracerebroventricular administration after SAH induction. The rats were randomly divided into four groups: sham group, SAH + vehicle (0.9% sterile saline, intraperitoneal administration) + vehicle (dimethyl sulfoxide, intracerebroventricular injection) group, SAH + NaHS (5.6 mg/kg, intraperitoneal administration) + vehicle (dimethyl sulfoxide, intracerebroventricular injection) group, and SAH + NaHS (5.6 mg/kg, intraperitoneal administration) + chelerythrine (1 mmol/L, 10 µl, intracerebroventricular injection) group. The expression of ROS and target proteins was tested at 24 hours after SAH induction. Twelve rats per group were separately sacrificed for ROS evaluation (*n* = 6 for each group) and proteins detection, including MST1, cl-MST1, Bcl-2, Bax, and caspase 3 (*n* = 6 for each group).

### Neurobehavioral function and severity of SAH assessments

Neurobehavioral function was blindly assessed by the Garcia Scoring System with modifications based on previous reports [35]. Briefly, this Garcia Scoring System contains six test items: spontaneous activity (0-3), climbing (1-3), forelimb stretching (0-3), spontaneous movements of all limbs (0-3), body proprioception (1-3), and response to vibrissae touch (1-3). Total scores range from 3 to 18, which parallels the severity of neurological dysfunction. A lower score indicates a worse neurological function induced by SAH. The SAH grade was performed to estimate the amount of bleeding in the subarachnoid space around the basilar artery rings and brainstem, as described in a previous study [35]. Total scores range from 3 to 18, in which a higher score indicates more serious SAH. In the present study, we only included those rats with an SAH grade score ≥ 8 at 24 hours after SAH induction.

### Brain water content

Brain edema was assessed by measuring brain water content according to previous studies [35]. The rats were sacrificed at 24 hours after SAH induction. The brains were removed and separated into four parts: left hemisphere, right hemisphere, cerebellum, and brain stem. Each part was immediately weighed to obtain the wet weight; these parts of the brain were then dried at 105 °C for 72 hours to obtain the dry weight. The brain content was defined as follows: brain content = (wet weight−dry weight)/wet weight×100%.

### Assay for ROS expression

Both SAH and sham rats were sacrificed at 24 h after endovascular perforation. Brain tissue samples around the basal cortical area in the injured side were obtained for assessment. Immediately after, the total ROS was measured by the ROS/RNS assay kit (Cell Biolabs, Inc., USA), following the manufacturer’s instruction.

### Western blotting

Six rats per group were sacrificed at 24 h after SAH induction or sham operation. Brain tissue samples were obtained from the same part of the left basal cortical area. Western blotting was performed as previously described [36]. Proteins were extracted from the brain tissue samples by RIPA buffer (Santa Cruz Biotechnology, CA, USA). Primary antibodies that recognized MST1 (Cell Signaling Technology, CST#3682), active caspase 3 (Abcam, ab49822), Bcl-2 (Cell Signaling Technology, CST#2876), and Bax (Santa Cruz Biotechnology, SC-493) were used.

### Immunofluorescence staining

Rats were perfused with PBS followed by 4% paraformaldehyde. The whole brain was removed, immersed in 4% paraformaldehyde for 24 hours, and transferred to a 30% sucrose solution for dehydration. Subsequently, the brain samples were frozen and cut into coronal frozen slices (slice thickness: 8 μm) with a cryostat microtome (Leica CM3050S-3-1-1, Bannockburn, IL). The sections were incubated overnight at 4 °C with primary antibodies, including those against MST1 (BioLegend, 611052), NeuN (Abcam, ab177487), GFAP (Millipore, ab5804), and IBA-1 (Abcam, ab5076), followed by the appropriate fluorescence-conjugated secondary antibodies (Jackson ImmunoResearch, West Grove, PA). TUNEL staining was performed according to the protocol from the manufacturer (Roche Inc, Basel, Switzerland) as described in previous studies [36]. The sections were visualized with a fluorescence microscope (LSM-710; Zeiss, Oberkochen, Germany). The percent of TUNEL-positive neurons was calculated in a blinded manner.

### Statistical analysis

All data are presented as the means ± standard error (SEM). One-way ANOVA analysis of the mean values, followed by an LSD test, was performed for multiple groups. A Mann-Whitney U test was used for the Garcia score and the SAH grading score. All of the analyses were performed using SPSS version 22.0 (SPSS Inc.). Statistical significance was defined as *P* < 0.05.

## Abbreviations

ROS: reactive oxygen species
MST1: mammalian sterile 20-like kinase 1
H_2_S: hydrogen sulfide
SAH: subarachnoid hemorrhage
NaHS: sodium hydrosulfide
cl-MST1: cleaved Mammalian Sterile 20-like Kinase 1;
EBI: early brain injury
CBS: cystathionine-β-synthase
3MST: 3-mercaptopyruvate sulfur transferase
